# Rider education at Swedish riding schools: Comparing teachers' and pupils' perspectives

**DOI:** 10.1371/journal.pone.0331059

**Published:** 2026-02-27

**Authors:** Lina Nyberg, Mari Zetterqvist Blokhuis, Andrew McLean, Elke Hartmann

**Affiliations:** 1 University of Helsinki, Faculty of Educational Sciences, Helsinki, Finland; 2 MZ Equitation, Skokloster, Sweden; 3 Equitation Science International (ESI), Victoria, Australia; 4 Department of Applied Animal Science and Welfare, Swedish University of Agricultural Sciences, Uppsala, Sweden; Universidade Federal de Mato Grosso do Sul, BRAZIL

## Abstract

Previous research has highlighted a gap between scientific evidence and its application in equestrian practice, leading to concerns about horse welfare and human safety. Riding schools present an important platform for promoting science-based practices, as they bring together riders of all ages and levels and serve as sites for equestrian education and for shaping attitudes toward horse welfare. Yet, the teaching in riding schools is often rooted in traditional practices. Therefore, mapping current educational methods and exploring how teaching and learning are perceived by both riding school teachers and pupils are key to supporting schools in bridging the gap between tradition and evidence-based practices. This study aimed to map how equestrian knowledge, with a specific focus on horse behaviour and welfare (BW), and horse learning and human-horse communication (LC), is taught and perceived to be learned, drawing on the perspectives of both teachers and pupils. Data collection was based on two online surveys, distributed to approximately 450 Swedish riding schools (RS) under the Swedish Equestrian Federation. The survey links were shared via the Federation’s newsletter, as well as through social media, horse magazines, and relevant equestrian websites. The surveys collected responses from 199 teachers and 368 pupils. The results showed that most teachers (83%) integrated BW and LC into regular riding lessons and 59% provided such education outside riding lessons. Yet, only 21% of the teachers believed that pupils learn enough when BW and LC are taught in connection with other teaching occasions. While 71% of pupils expressed interest in dedicated BW and LC lessons, only 24% of teachers thought pupils were interested in attending. The main barrier to offering separate lessons, according to teachers, was perceived lack of interest (50%), whereas 30% of pupils cited the unavailability of such lessons. These differing perceptions highlight the need for improved communication and greater alignment between teaching practices and what learners find engaging. The findings from this study offer a foundation for developing strategies to better support evidence-based equestrian education at riding schools.

## Introduction

In recent decades, growing concern for horse welfare has prompted a shift away from traditional explanations and approaches to equestrian practices toward a more evidence-based understanding of human-horse interactions. This shift is largely grounded in the fields of equitation science, animal welfare science, and human-animal studies, all of which offer valuable frameworks for examining how equestrian knowledge is taught and applied in educational settings like riding schools.

Equitation science [1] refers to the systematic study of horse-human interactions, with the aim of providing objective, evidence-based explanations for best practices that enhance horse welfare and human safety, while identifying training methods that may jeopardise these outcomes [2]. Likewise, contemporary animal welfare science offers a horse-centred perspective, focusing on the individual’s subjective lived experience and offering structured tools, such as the Five Domains Model [3], for assessing welfare across physical, environmental, and social dimensions [4]. Meanwhile, social sciences, particularly human-animal studies, contribute important insights into the social, ethical, and cultural dimensions of equestrian practices and interspecies relationships [5]. For example, natural horsemanship enthusiasts may shift between scientific explanations of horse behaviour, drawn from ethology, to portraying horses as partners and almost human, reflecting contradictions of the way they talk about horses [6]. Other authors describe the human-horse relationship as involving bodily ways of knowing that go beyond verbal communication, challenging traditional research methods and offering new perspectives on how knowledge is shared between species [7]. This social science approach to studying human-animal relationships is also increasingly used to explore how equestrian knowledge is constructed, transmitted, and interpreted within specific educational settings such as in the coaching of riders [8–11] and in the teaching of young children at riding schools [12,13].

Knowledge and skills have been argued to be key components of human behaviour change with beneficial effects on animal welfare, and horse welfare in particular [14,15]. However, scientific evidence indicates that many individuals involved in equestrian activities do not have sufficient equine knowledge or overestimate their own horse-related knowledge, a phenomenon consistent with the Dunning-Kruger effect [16]. This may lead to inappropriate management and training methods with negative consequences for horse welfare. For example, the conceptualisation of horse welfare among amateur equestrians does not align with neither the Five Domains Model nor with what riders do in practice [17]. Further, it is not uncommon for equestrians to fail to recognise signs of stress, such as interpreting a horse’s forwardness or high energy as expressions of “happiness” [18]. On this note, it has recently been suggested that professional trainers’ teaching methods may not adequately support riders in developing a practical understanding of horse behaviour, as this aspect is not systematically embedded into their teaching [19]. Other studies have shown that knowledge of learning theory as applied to horse training [20] is relatively low among both amateur and professional riders [21,22]. This lack of understanding increases the risk of misunderstandings, ineffective training, and the use of coercive methods, all of which compromise horse welfare and negatively impact rider safety. This, together with poor horse welfare, has been connected to high numbers of human accidents [23,24]. Despite advancements in rider safety equipment, horse sports continue to rank among the most dangerous sporting activities globally [25]. Although equine-related activities can possibly never be entirely safe, acquisition of appropriate knowledge of how to manage and train horses is likely to prevent accidents and injuries [26,27].

Without doubt, there is a need for a universal cultural shift towards an evidence-based approach to training horses, with equine welfare at its core, as recently highlighted by workshop participants in a study by Thorell Palmquist et al. [28]. The authors further suggest that educational institutions are well-positioned to lead this change and call for research that addresses pedagogical challenges. Riding schools are well positioned to promote an education that aligns with scientific advances in knowledge of horse behaviour, welfare, learning, and human-horse communication, with riding teachers playing a central role in challenging traditional views. Yet, despite this potential, traditional teaching practices seem to continue to dominate equestrian education [29,30] where focus remains on communicating the technical aspects of riding, such as how to sit on the horse or how the horse moves in response to the riders’ aids [8,10]. Little attention is paid to developing the riders’ ability to interpret horses’ behavioural and emotional responses [19,31,32] despite existing protocols that could ensure an objective and more consistent interpretation of horses’ reactions [33–35]. This limited integration of science-based content is further evidenced by recent survey data showing that only 36% of the Swedish and Finnish pupils reported that non-riding education in the subjects of horse behaviour, welfare, learning, and human-horse communication is offered at their riding schools [36]. Although it was not specified whether non-riding refers to theory and/or in-hand (ground-work) lessons, the latter is known to provide numerous benefits, such as teaching riders safe horse handling and observational skills [1]. Further, behavioural responses in-hand can give direct feedback on the quality of responses the horse provides under saddle [1,37]. To become a competent rider, it has been suggested that a combination of theoretical knowledge, technical skill, and practical wisdom is essential. Developing such wisdom also requires experience and the ability to reflect on that experience. Yet, structured reflection is not sufficiently embedded in the riding school curriculum [31].

In Sweden, there are around 450 riding schools that are mainly run by non-profit riding clubs affiliated with the Swedish Equestrian Federation [30,38]. Each year, these schools provide approximately five million riding lessons to beginners and advanced riders of all age groups [39]. The aim of this study was to identify the current state of theoretical and practical education in the subjects of horse behaviour, welfare, learning, and rider-horse communication and explore how this education, or the lack thereof, is perceived by both teachers and pupils as well as to identify obstacles to providing and attending such education.

## Materials and methods

### Ethical statement

According to the Swedish Ethical Review Act (2003:460, Swedish Ethical Review Authority, https://etikprovningsmyndigheten.se/en/; [40]), this type of online survey study did not require ethical approval. This was because no sensitive personal data were collected, no identifiable or sensitive information was processed, all responses were anonymous, and none of the questions were of a nature likely to influence participants physically or mentally. Participants were fully informed about the study’s purpose, main contact person of the research, and their right to withdraw at any time via written information provided on the opening page of the web survey. Consent was obtained prior to participation, following standard practice for online surveys, by participants clicking an ‘I agree’ button before proceeding. Participation of pupils aged 15–18 also fell outside the scope of ethical review, as individuals in this age group can provide their own consent without guardian approval according to § 18 of the Ethical Review Act.

### Questionnaire design

Data collection was based on a sample of volunteer participants from the Swedish equestrian community, who completed two online questionnaires concerning educational practices within Swedish riding schools and their perception of them. The questions were designed to address two main subject areas: Behaviour and Welfare (BW) and Learning and Communication (LC). These subjects were selected because knowledge of BW and LC can improve the welfare and safety of horses and riders, while also enhancing training effectiveness and promoting ethical practices [1]. Questionnaire 1 (total 42 questions) gathered perspectives from riding school teachers, and questionnaire 2 (total 34 questions) collected responses from pupils taking riding lessons. The term ‘teacher’ is used throughout this article to refer to respondents in an instructional role, even though their formal educational backgrounds as riding instructors may vary. Hence, all respondents in this category were asked to respond based on their role as a teacher, i.e., someone providing mounted and/or unmounted lessons to riders.

The questionnaires consisted of four sections: demographics, teaching methods, perceived knowledge gain, and interest in, as well as reported barriers to, attending subject teaching. Respondents were asked to refer to the riding school they were currently teaching at or attending for lessons during the spring term 2022. The questionnaire was set up so that respondents were directed toward relevant questions based on their previous answers. For example, if respondents answered that they did not provide separate, non-riding lessons (teacher) in the subjects BW and/or LC or did not attend such lessons (pupils), they would skip further detailed questions concerning teaching practices and opinions related to it.

The questionnaires predominantly consisted of closed-ended questions with predetermined response categories, supplemented in some cases by an ‘other’ option allowing respondents to provide their own defined category or comment openly. Likert scales were used to measure the degree of agreement or disagreement with statements concerning opinions and knowledge base with 1 indicating strong disagreement and 5 indicating strong agreement.

Interviews were conducted with three riding school teachers and two riding school pupils to aid in drafting the questions. The questionnaires were then piloted with members of the project group (i.e., riding teachers, researchers in equine science and social sciences) and riding school pupils not subsequently involved in the study.

### Demographics

All respondents were asked to report their age, gender, years of teaching experience (teachers), and years of experience with horses (pupils). Teachers were also asked to report their level of education (answer options: *riding teacher at level 1–3*; *licenced trainer at level A-C*; *other education; no education*), and number of lessons taught per week. Pupils reported the number of riding lessons attended weekly.

To get an overview of the geographical distribution of riding schools in Sweden, both teachers and pupils were asked to indicate the region in which their school was located (*Norrland*; *Svealand*; *Götaland*). Notably, this regional data was not specific enough to identify individual schools.

### Teaching methods and subject knowledge

Teachers were asked to report whether, in addition to regular taught riding lessons, they had provided separate non-riding lessons solely focusing on BW and/or LC during the spring term 2022 (*yes*; *no*). Pupils were requested to indicate whether they had participated in such lessons during the same term (*yes*; *no*; *not provided for my age group*; *not provided at the school*). Further, both teachers and pupils were asked if they perceived that these subjects were integrated in regular riding lessons and/or taught in conjunction with other teaching occasions (*yes – during ridden education including tacking-up and general horse care; yes* – *during other education such as ground-work*, *theory lessons, lessons in the stable; no*). As a follow-up, teachers were asked if they perceive that pupils learn enough, and pupils were asked if they feel they learn enough if BW and LC are taught in conjunction with other teaching (*yes; no; don’t know/no opinion*).

Moreover, pupils were asked if they had any opportunities to influence the teaching of the two subject areas with the following answer options: *yes – I can come with own suggestions*; *teachers’ own knowledge and wishes dominate*; *school follows an established educational plan*; *no – I cannot influence the teaching*; or *other*. As opposed to pupils, teachers were requested to specify the criteria they use when selecting which subjects are taught: *joint planning with other teachers*; *according to pupils’ wishes*; *according to teachers’ knowledge base and wishes*; *according to the school’s educational plan*; *according to the riding-handbooks*; or *other*.

### Perceived knowledge gain and opportunities for further learning

This section of the questionnaire contained queries regarding respondents’ perceived level of knowledge, current sources of knowledge acquisition, and suggestions for additional learning resources. Teachers were asked to rate how they perceive pupils’ theoretical and practical understanding in the two subjects on a scale from 1 (*no knowledge at all*) to 5 (*extensive knowledge*). A similar question was directed to pupils, assessing their satisfaction with their own level of knowledge (*1 – not at all satisfied* to *5 – very satisfied*). The same question was also presented to teachers. Respondents were further asked if they want to improve their subject knowledge (*yes*; *no*) and if so, how they would like to improve it by giving them multiple predefined options: *further education to achieve a higher level certification for teachers/trainers or riding badges for pupils*; *continued training that complements my current education such as folk high school or tertiary education*; *home studies such as attendance of clinics, webinars*; *written information in format of, e.g., newsletter, information sheet*; *more practical experience*. For pupils, the answer option ‘*possibility for more education at the riding school*’ was added to this list.

Moreover, current sources of knowledge acquisition were investigated, enabling every respondent to pinpoint three out of fourteen sources they consider to be most significant: *website of the Swedish Equestrian Federation*; *website of ‘HästSverige’* (Swedish online platform that translates scientific findings into lay language); *other websites*; *popular equestrian journals*; *scientific articles*; *books*; *social media*; *blogs and/or forums*; *films (e.g., on YouTube)*; *friends and/or equestrian friends*; *other riding teachers and/or trainers*; *veterinarians and/or farriers*; *researchers*; *intuition and/or common sense*. As in the previous section of the questionnaire, respondents could always choose the answer option ‘*no opinion*’ or specify with their own words an alternative category ‘*other’*.

### Interest and barriers to subject teaching and learning

In the final section of the questionnaire, data on respondents’ opinions and attitudes towards the teaching of the subjects were assessed. Teachers were prompted to provide feedback on their perception of the general level of pupils’ interest in the two subject areas BW and LC on a Likert scale from *1 (not at all interested)* to *5 (very interested*). A follow-up question sought teachers’ perspectives on the level of pupils’ interest in pursuing additional education in these two subject areas at their riding school (*1 – not at all interested to 5 – very interested*) while pupils were asked to express their desire to attend such education, utilizing the same Likert scale as for teachers.

Further, both teachers and pupils were asked if they experience any obstacles in providing lessons in the subjects and in attending such lessons, respectively. Teachers could choose one or several of the following options: *lack of time; lack of pupils’ interest; lack of own knowledge and/or experience; lack of educational material; it is not economically viable for the riding school; no obstacles*). The answer choices were adapted to reflect the viewpoints of pupils, allowing them to select their responses accordingly: *no lessons offered at the riding school; no lessons offered at the school for my age class; lack of time; economic reasons; teachers’ lack of interest and competence; own lack of interest; lack of educational material; no obstacles*.

Finally, both teachers and pupils were inquired as to their level of satisfaction with the teaching provided in the two subject areas of equine knowledge, irrespective of whether the teaching occurred through tailored lessons or in conjunction with riding (*1 – not at all satisfied to 5 – very satisfied*). In all opinion-based questions, respondents were provided the choice to select ‘*no opinion*’ or ‘*other*’, providing them with the opportunity to offer additional details or explanations for their response.

### Questionnaire distribution

The questionnaires were created with the online survey tool Netigate (Netigate AB, Stockholm, Sweden). The Swedish Equestrian Federation disseminated the links to the two questionnaires, along with information about the study’s objectives, via two newsletters sent to around 450 registered riding schools in Sweden. The questionnaire links were also distributed via the online platform HästSverige, social media, equestrian magazines such as Hippson, and Tidningen Ridsport, and through personal contacts with heads of riding schools. The questionnaires were available online during May and June 2022, and respondents were asked to relate their answers to the current spring term at their riding school. Riding teachers from the age of 18 and riding school pupils from the age of 15 were invited to take part in this study.

### Statistical analysis

Raw data were exported from the online survey tool Netigate as an Excel spreadsheet. Descriptive statistics were calculated in Excel (version 2016), and further statistical analyses were performed using Minitab Statistical Software (version 19.2020.1). Only fully completed surveys were included in the analysis.

For all Likert-scale questions, responses were recoded to reduce the number of categories from five to three whereby answer options 1–2 and options 4–5 were combined. Responses marked as ‘no opinion’ were excluded from the analysis when counts were below five; however, removing these did not affect the results and was not applied systematically across all statistical tests.

Cross-tabulations were created to display the distribution of responses, and nonparametric Pearson chi-squared tests were conducted to examine differences between groups (teachers vs. pupils) and between the two subject areas BW and LC. Statistical significance was set at p < 0.05.

## Results

The survey generated data from 387 riding school teachers and 589 riding school pupils, of which 199 and 368 completed the survey, respectively.

### Demographics

The majority of respondents were female (95%), and the most represented age group was 46–55 years of age (see Table 1). Over half of the teachers had more than 16 years of teaching experience (58.3%, 116/199) and 47.3% (174/368) of pupils reported having 16 or more years of riding experience (Table 1). Most of the teachers had a formal education (62.3%, 124/199), corresponding to the Swedish certified riding instructor levels I (12.6%, 25/199), II (32.7%, 65/199), and highest III (17.1%, 34/199). A total of 12.6% (25/199) of the teachers reported that their educational background was as a ‘riding leader,’ a position regarded as the entry-level qualification for teaching at a riding school before advancing to certification as a riding instructor under the Swedish Equestrian Federation guidelines. Two respondents specified being a licenced level B and C trainer, one a Swedish fellow. Sixteen percent (32/199) chose the answer category ‘other’ and specified their education openly (e.g., certified groom, youth leader, certified trainer in academic art of riding, equine science education, animal trainer and nurse, ethologist). The answer option ‘no formal education’ was chosen by 8% of respondents (16/199).

**Table 1:** Riding school teachers’ (N = 199) and pupils’ (N = 368) demographics, presented in percentage (and number) of respondents.

Question / Catgeory	Teacher	Pupil
**Gender**		
Woman	98.5 (196)	95.7 (352)
Man	1.0 (2)	2.7 (10)
Other	0.5 (1)	0.3 (1)
Prefer not to say	0.0 (0)	1.4 (5)
**Age in years** ^**1**^		
15-25	7.0 (14)	23.4 (86)
26-35	17.6 (35)	13.6 (50)
36-45	26.1 (52)	19.6 (72)
46-55	33.2 (66)	26.1 (96)
> 55	16.1 (32)	17.4 (64)
**Experience in years** ^**2**^		
< 5	15.6 (31)	14.4 (53)
6-10	11.6 (23)	19.0 (70)
11-15	14.6 (29)	17.4 (64)
16-20	16.1 (32)	11.4 (42)
> 20	42.2 (84)	35.9 (132)

^1^Teacher respondents’ minimum age for participation was 18 years old.

^2^Corresponds to years of teaching (teacher) versus years of riding (pupils).

Twelve percent of the teachers (23/199) answered to give between 1–4 hours of lessons per week at the school. Most teachers replied that they were providing between 5–10 riding lessons (42.2%, 84/199) and 11–20 lessons (42.2%, 84/199) per week. Only 4% (8/199) of the teachers reported to teach more than 20 hours weekly. Most pupils took one lesson weekly (57.6%, 212/368), followed by riding 2–3 times per week (34.8%, 128/368). The minority of pupils reported riding less than once weekly (7.1%, 26/368) and two respondents reported to attend more than 4 lessons per week (0.5%, 2/368).

According to both teachers and pupils, most riding schools were located in ‘Svealand’ (53.3%, 302/567) in central Sweden. Götaland, in the south, accounted for 28.9% (164/567), while 17.8% were situated in northern Norrland (101/567).

### Teaching methods and subject knowledge

**Fig 1:**
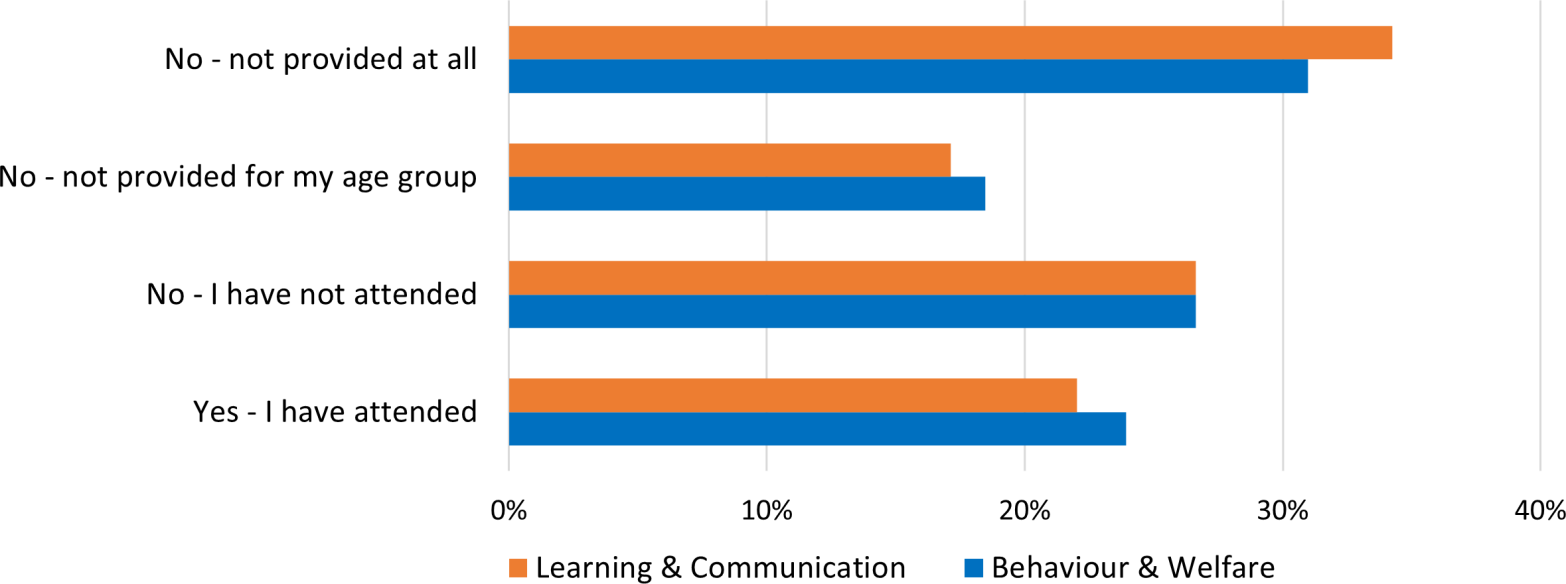
Overview of pupils’ replies (N = 368) on attendance of separate non-riding lessons in the two equine subject areas.

Among the pupils, 23.9% (88/368) had attended unmounted lessons solely dedicated to teaching BW, and 22% (81/368) in LC during the spring term 2022. Nearly a third (BW: 30.1%, 112/368; LC: 33.7%, 124/368) indicated that separate lessons were not provided at all at their riding school (see Fig 1). Most teachers reported providing separate non-riding lessons focusing on BW (64.3%, 128/199) and LC (52.8%, 105/199).

Chi-square tests revealed significant associations between teachers and pupils and their perceptions of whether BW and LC were included in the riding school education during riding and/or un-mounted lessons, including general care of the horse. Teachers consistently reported higher inclusion of these subjects compared to pupils, both during riding lessons and unmounted lessons. Conversely, pupils more frequently indicated that BW and LC were not taught (see Table 2). These associations were significant for both BW (Χ^2^ = 66.61, df = 2, p < 0.001) and LC (Χ ^2^ = 43.24, df = 2, p < 0.001).

**Table 2:** Responses of teachers (N = 199) and pupils (N = 368) regarding the incorporation of the two equine subjects into regular teaching, presented in percentage (and number). Respondents had the option to select multiple answers.

Subject	Behaviour & Welfare		Learning & Communication	
**Category**	**Teacher**	**Pupil**	**Teacher**	**Pupil**
During riding lessons^1^	82.4 (164)	46.5 (171)	84.4 (168)	41.9 (154)
During unmounted lessons^2^	77.4 (154)	50.0 (184)	70.9 (141)	37.8 (139)
Not taught	3.5 (7)	28.3 (104)	6.0 (12)	20.4 (75)
Total	325	459	321	368

^1^Can include tacking-up and general horse care prior to riding lessons.

^2^Can include working horses from the ground and/or theory lessons.

Statistically significant associations were found between teachers’ and pupils’ perceptions of whether pupils learn sufficiently about BW and LC when taught in conjunction with other teaching occasions. Pupils were more likely than teachers to report that they learn enough in these subject areas, while teachers were more sceptical, responding more often with ‘no’ or ‘no opinion’. For example, 37.8% (139/368) of pupils felt that they were learning enough about BW, compared to 22.1% (44/199) of teachers who believed this was the case. Similarly, 38.3% (141/368) of pupils said they learn enough about LC, versus 20.1% (40/199) of teachers. Notably, the majority of teachers (71.9%, 143/199) felt that pupils do not acquire sufficient knowledge about BW when it is taught as part of riding lessons or other teaching, with a similar trend observed for LC. These differences in perceptions were statistically significant for both BW (Χ ² = 49.36, df = 2, p < 0.001) and LC (Χ ² = 40.40, df = 2, p < 0.001).

**Fig 2:**
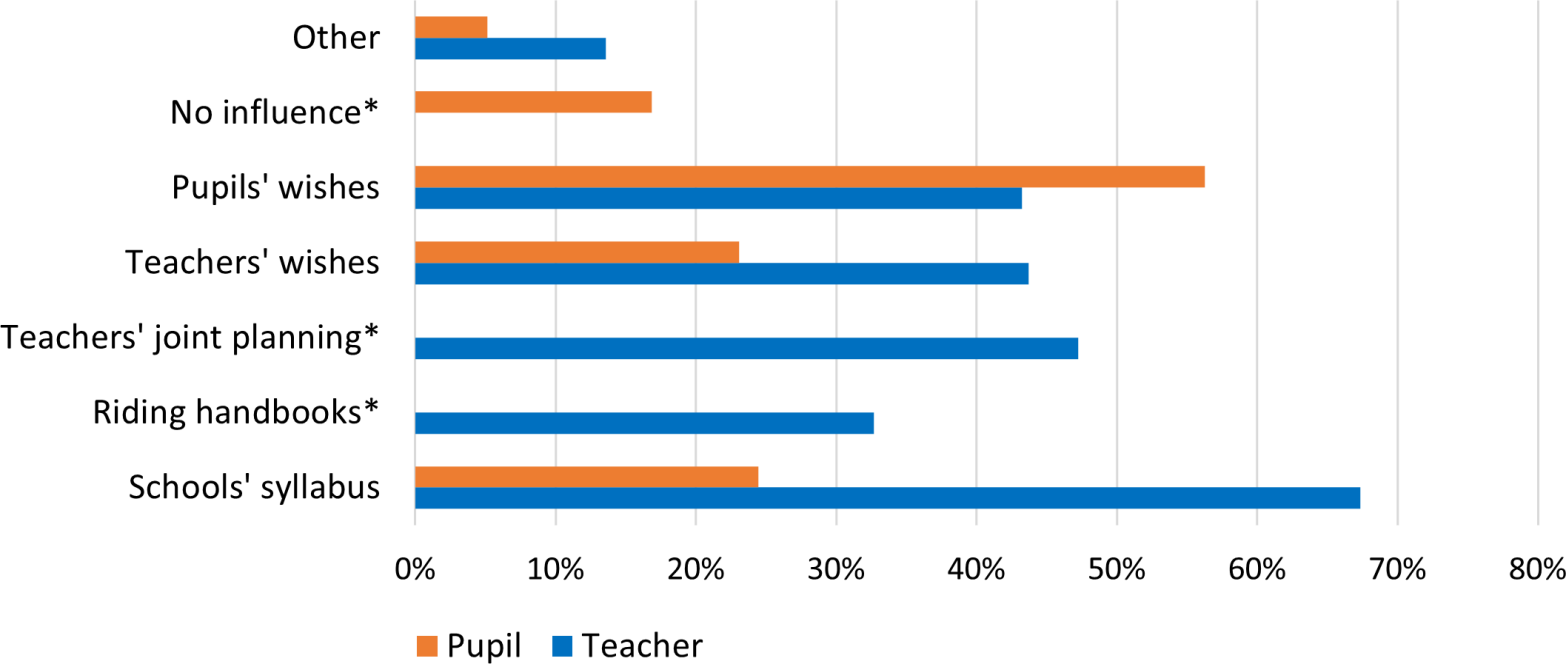
Overview of teachers’ (N = 199) and pupils’ replies (N = 368) on their perceived ability to influence the teaching in the two subjects. All respondents could select multiple answer options. Items marked with an asterisk (*) indicate response options that were not provided to either teachers or pupils, depending on relevance.

When pupils were asked about their possibilities to influence the teaching of both subjects, the majority indicated they could express their own preferences (56.3%, 207/368), and over 40% (86/199) of teachers considered pupils’ wishes (see Fig 2). Most teachers reported primarily following the school’s syllabus (67.3%, 134/199) and making decisions about subject teaching through collaborative planning with other teachers (47.2%, 94/199).

### Perceived knowledge gain and opportunities for further learning

Almost all teachers (97.5%, 194/199) considered that it is important or very important for riding schools to disseminate evidence-based knowledge in the two subject areas BW and LC. None of the teachers thought it was not of any importance. Less than 50% of the teachers indicated that the implementation of evidence-based knowledge in the teaching at riding schools is easy (42.7%, 85/199). Thirty-five percent (34.7%, 69/199) of teachers thought it was neither difficult nor easy, and 17.6% (35/199) replied that it was difficult. Five percent of teachers chose the answer option ‘no opinion’ (10/199).

The majority of respondents were satisfied with their own subject knowledge. However, teachers reported higher satisfaction levels with their own knowledge compared to pupils, a trend observed in both BW (Teacher: 85.9%, 171/199; Pupil: 68.8%, 253/368) and LC (Teacher: 84.4%, 168/199; Pupil: 66.0%, 243/368). Pupils more frequently reported being unsatisfied with their own knowledge in both subject areas (BW: 8.7%, 32/368; LC: 9%, 33/368) compared to teachers (BW: 1.5%, 3/199; LC: 1.5%, 3/199). Chi-square test showed that these trends were statistically significant both for BW (Χ ² = 21.10, df = 2, p < 0.001) and LC (Χ ² = 23.37, df = 2, p < 0.001).

Teachers were asked to estimate how much practical and theoretical knowledge they believe pupils have in the two equine subject areas. In BW, 34.7% (69/199) were dissatisfied with pupils’ knowledge, 43.2% (86/199) responded neutrally, and 20.6% (41/199) were satisfied. In LC, 40.2% (80/199) of the teachers indicated that they were unsatisfied, 43.2% (86/199) neutral, and only 15.1% (30/199) satisfied. In both subjects, 1.5% (3/199) selected ‘no opinion’.

**Fig 3:**
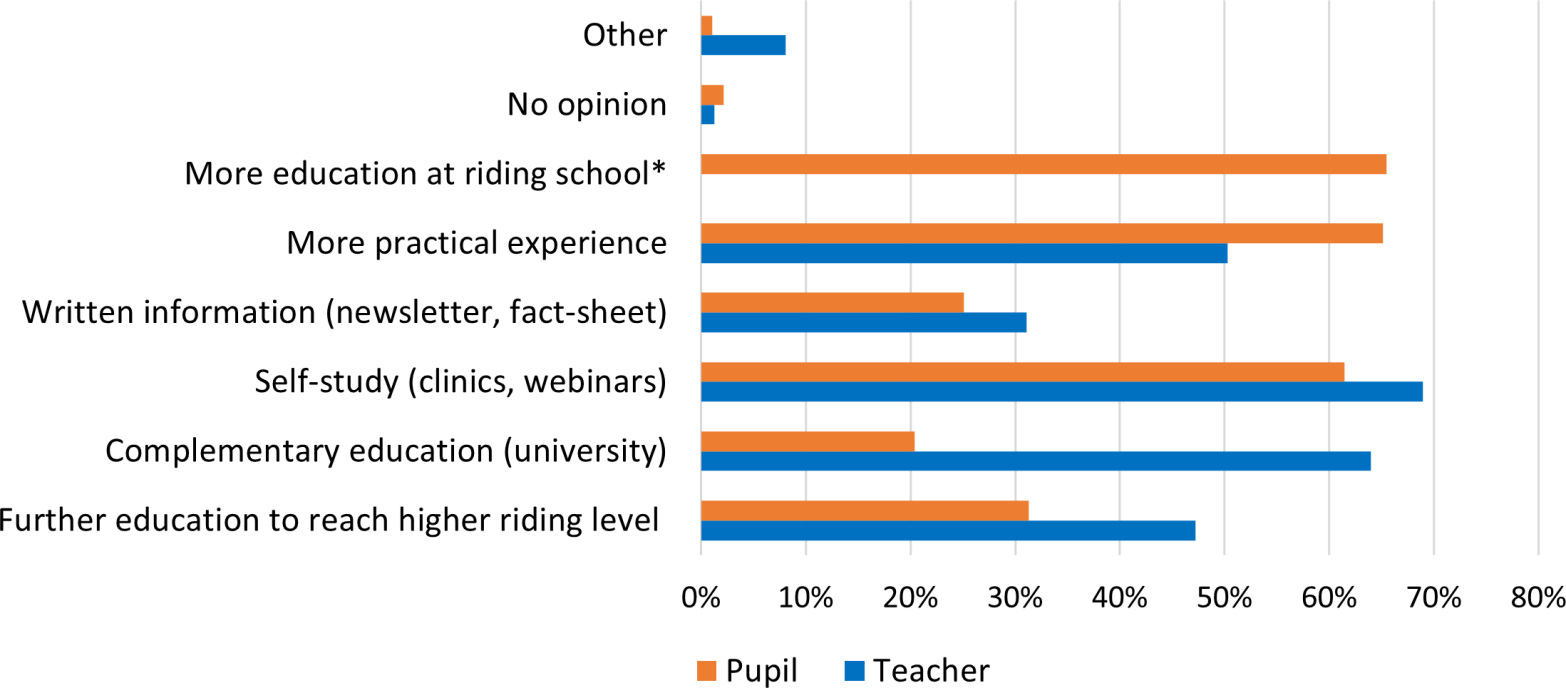
Teachers’ (N = 161) and pupils’ (N = 275) responses on formats of further knowledge gain. Respondents could select multiple answer options. Items marked with an asterisk (*) indicate a response option only provided for pupils.

Both teachers and pupils were asked whether they wished to improve their own subject knowledge. A majority in both groups expressed a desire to do so, with 80.9% of teachers (161/199) and 74.7% of pupils (275/368) responding ‘yes’. Chi-square test showed no statistically significant association between groups (Χ ² = 3.42, df = 2, p = 0.181), indicating a similar distribution of responses across both groups. A follow up question asked about preferred formats for gaining additional subject knowledge. Pupils expressed a strong desire for more teaching offered at the riding schools (65.5%, 180/275) and for increased opportunities to gain practical experience (65.1%, 179/275) (see Fig 3). A large proportion of both teachers and pupils indicated they also wish to improve their knowledge base through self-study, such as through participation in clinics or webinars (Teacher: 68.9%, 111/161; Pupil: 61.5%, 169/275).

**Fig 4:**
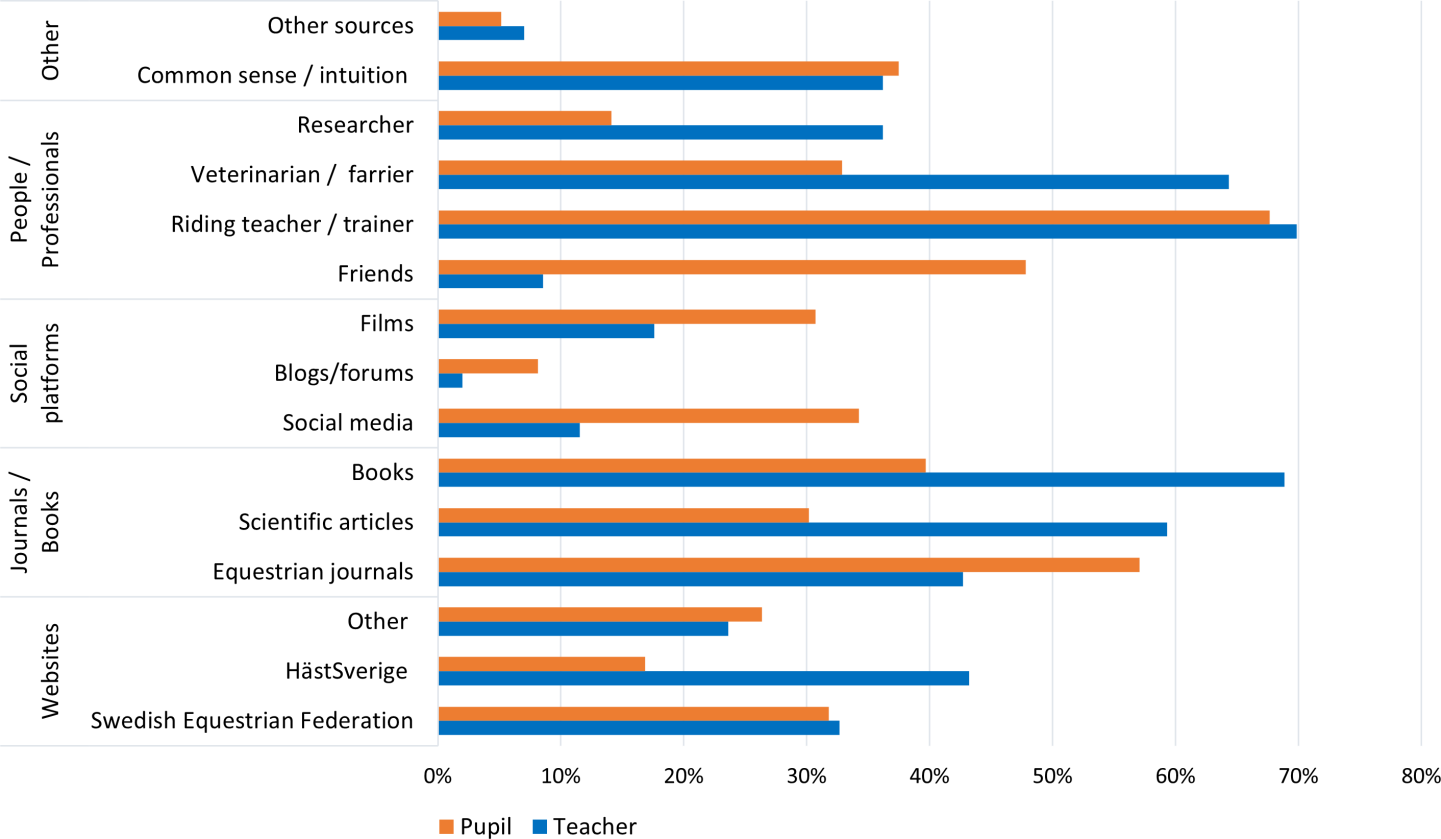
Teachers’ (N = 199) and pupils’ (N = 368) responses on preferred sources of knowledge gain. Respondents could select from multiple sources.

Fig 4 provides an overview of various sources teachers and pupils specified to utilize for own knowledge acquisition. The majority of teachers reported to turn towards colleagues (69.9%, 139/199), books (68.8%, 137/199), and veterinarians and/or farriers (64.3%, 128/199). Pupils reported to rely mostly on their riding teacher and/or trainer (67.7%, 249/368), equestrian journals (57%, 208/368), and friends (48%, 174/368).

### Interest in, and barriers to, teaching and learning

For both BW and LC, 36.7% of teachers (73/199) believed that pupils were interested in the subject areas. However, an equally large proportion selected the neutral option for BW (36.7%, 73/199), and an even higher proportion did so for LC (38.7%, 77/199), indicating that many teachers were uncertain about pupils’ level of interest. A smaller percentage considered pupils as uninterested (BW: 24.6%, 49/199; LC: 23.6%, 47/199), while 2.0% (4/199) selected ‘no opinion’ for both subjects.

A follow-up question investigated teachers’ opinions on how interested they thought pupils are in attending additional subject teaching. The same question was provided to pupils asking whether they would attend such education. There was a significant association between teachers’ and pupils’ interest in attending BW (Χ = 99.48, df = 3, p < 0.001) and LC (Χ ^2^ = 138.17, df = 3, p < 0.001) lessons. Pupils reported a substantially higher level of interest in both subject areas compared to what teachers believed. In BW, 67.1% of pupils (247/368) indicated they were interested in attending additional lessons, compared to 23.6% (47/199) of teachers who believed this to be the case. Similarly, in LC, 75.7% of pupils (255/337) expressed interest, while only 23.6% (47/199) of teachers perceived pupils as interested.

Another opinion question inquired about how satisfied both teachers and pupils were with the provided teaching in the subject areas. Statistically significant associations were found for both BW (Χ ² = 37.93, df = 3, p < 0.001) and LC (Χ ² = 32.26, df = 3, p < 0.001). In BW, 66.8% of teachers (133/199) reported being satisfied, compared to 43.5% of pupils (160/368). Similarly, for LC, 62.8% of teachers (125/199) reported satisfaction compared to 43.2% of pupils (159/368) (see Table 3).

**Table 3:** Teachers’ (N = 199) and pupils’ (N = 368) responses on the general level of perceived interest in attending additional offered subject lessons and the level of satisfaction with the current teaching.

Subject	Behaviour & Welfare		Learning & Communication	
**Category**	Teacher	Pupil	Teacher	Pupil
*Interest in attendance* ^*1*^				
Uninterested	37.7 (75)	13.9 (51)	34.7 (69)	10.4 (35)
Neutral	33.7 (67)	16.3 (60)	36.7 (73)	11.9 (40)
Interested	23.6 (47)	67.1 (247)	23.6 (47)	75.7 (255)
No opinion	5.0 (10)	2.7 (10)	5.0 (10)	2.1 (7)
Total	199	368	199	337
*Satisfaction with teaching*				
Unsatisfied	11.1 (22)	17.4 (64)	13.1 (26)	18.8 (69)
Neutral	18.6 (37)	21.5 (79)	20.6 (41)	20.7 (76)
Satisfied	66.8 (133)	43.5 (160)	62.8 (125)	43.2 (159)
No opinion	3.5 (7)	17.7 (65)	3.5 (7)	17.4 (64)
Total	199	368	199	368

^1^Teachers indicated perceived interest of pupils. Pupils indicated perceived own interest.

**Fig 5:**
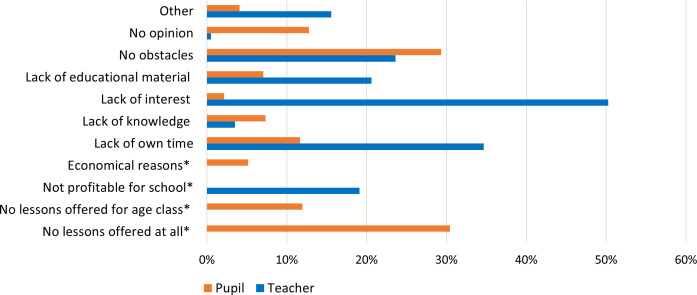
Teachers’ (N = 199) and pupils’ (N = 368) opinions on perceived obstacles in providing and attending, respectively, separate lessons in the two subject areas. Respondents could select multiple answer options. Items marked with an asterisk (*) indicate response options only provided for either category of respondent.

Further, teachers and pupils specified whether they experience any obstacles in providing lessons and in attending subject lessons, respectively. While such lessons were not provided according to most pupils (30.4%, 112/368), the majority did not experience any obstacles in attendance (29.4%, 108/368) (see Fig 5). According to teachers, the most frequently specified obstacle was a lack of interest among pupils (50.3%, 100/199), contrasting with pupils’ responses where only 2.2% (8/368) indicated a lack of interest. Teachers frequently cited a lack of their own time as another common obstacle (34.7%, 69/199), whereas pupils perceived a lack of time to a much lesser extent (11.7%, 43/368) (see Fig 5).

## Discussion

This study investigated the teaching and learning of equestrian knowledge in horse behaviour and welfare (BW) and horse learning and human-horse communication (LC) at Swedish riding schools from the perspectives of both riding school teachers and pupils. While previous research has recognized the need for increased knowledge and skills in these subjects, this study contributes new insights by focusing on how such education is currently delivered in a riding school context and perceived by those providing and taking part in the education. Our study findings add to existing research in equestrian education and suggest actionable steps for stakeholders to further develop more effective teaching practices at riding schools and the equestrian sector.

Overall, there were notable discrepancies between teachers’ and pupils’ perceptions of how the BW and LC subjects are taught at riding schools. A key finding was that BW and LC are predominantly taught in conjunction with riding lessons and/or other teaching occasions rather through standalone, non-riding education. Yet, pupils were less likely to perceive these subjects as being incorporated into broader teaching, expressed a greater desire to attend separate non-riding lessons than teachers expected, and were generally less satisfied with the current subject teaching than their teachers. All respondents expressed the desire to enhance their own subject knowledge in these areas with a notable majority of pupils interested in attending more of such education at the riding school. However, teachers reported several perceived barriers to delivering this education, especially a presumed lack of interest among pupils, an assumption not reflected in pupils’ responses. Clearly, the results revealed differences between teachers’ and pupils’ perceptions and satisfaction levels regarding the teaching of the two subjects. This highlights the importance of both understanding and addressing these discrepancies to better align teaching practices and pedagogical approaches with pupils’ interests and expectations, thereby promoting more effective learning experiences and outcomes [12].

In the following sections, the main findings are discussed, followed by a presentation of the limitations of the present study and implications for future research. For clarity and readability, the two subjects BW and LC are not considered separately in the discussion but are combined into one, as there were no major differences between them.

### Teaching methods and subject knowledge

In the present study, teachers more frequently reported offering separate non-riding lessons in the subjects during the spring semester 2022 than pupils confirmed attending such lessons during the same period. This discrepancy is consistent with recent findings from an online survey conducted by Nyberg et al. [36], in which only 26% of the Finnish and Swedish riding school pupils reported attending non-riding lessons in BW and LC during the last semester (spring 2022). One possible reason for the low attendance may be that such education is offered more often to beginner riders than to experienced riders. In line with previous findings [36], the majority of pupils in the present study had six or more years of experience with horses. Nevertheless, as previous research indicates, even experienced equestrians can lack adequate equine knowledge and may fail to recognize or correctly interpret horse behaviour, or may fail to apply learning theory appropriately to training in ways that promote positive experiences and good welfare [18,21,22,41–43]. This highlights the need for education in BW and LC across all experience levels.

Although most teachers perceived that BW and LC are taught in connection with riding lessons and/or other teaching, only a few believed that pupils learn enough when the subjects are integrated in this way. This result potentially suggests that the need for separate lessons in BW and LC is recognised among riding school teachers. Previous research within the equestrian teaching also indicates that theoretical knowledge [8] as well as unmounted practice [1] are important and should be an integral part of the education for equestrians. Yet, 32% of the pupil respondents in the present study reported that separate non-riding lessons in BW and LC are not provided at all at their riding school. Nonetheless, this suggests a somewhat more positive situation compared to Nyberg et al. [36], where 64% of the Finnish and Swedish pupils reported that separate unmounted education in the two subjects was either not provided or respondents were unsure of its availability. Considering that knowledge about horses and skills to manage and handle them is crucial for horse welfare [15,44] and human safety [23,24,45], these results point to a need for more structured education in BW and LC.

Moreover, better alignment between teaching and communication could enhance learning. In our study, significantly fewer pupils than teachers considered that BW and LC are taught in connection with other teaching sessions beyond riding, suggesting that pupils may not be aware of ongoing subject teaching during regular education. This points to a potential communication gap between teachers and pupils regarding instructional practices and intended learning outcomes. Importantly, alignment between teaching activities, intended learning outcomes, and assessment is acknowledged as a fundamental base for effective learning within the educational field [46]. For example, if the goal is to learn how to correctly interpret horse behaviour, the teaching should focus on that specific skill. Students should engage in the task and understand the intended outcome of the learning activity. Incongruence between teachers’ and pupils’ perceptions of education is not uncommon, but it is disadvantageous for the effectiveness of both teaching and learning [47,48]. Students who shared the least perceptions with teachers reported more emotional and motivational problems and performed worse [48], and students who failed to distinguish which tasks were important also failed to focus attention appropriately on the task or activity [47].

In this study, most teachers followed the riding school’s syllabus when deciding what to teach in BW and LC. This indicates that riding schools have a structured plan for teaching these subjects. However, it is unknown how much these plans differ between schools, how staff competencies influence the implementation, or to what extent the content aligns with current scientific advances in equine management, training, and pedagogics. Also, collaborative planning with other teachers was common in deciding on the teaching content in the two subjects, which is generally considered beneficial for both student learning and understanding, as well as teacher motivation and innovation [49]. On the other hand, collegial consensus may also hinder development if it maintains traditions of outdated practices [50]. Pupils in the present study reported that they can express wishes regarding the teaching of BW and LC, although fewer teachers considered pupils’ wishes. A feeling of being able to influence activities in which one participates is undoubtedly beneficial for pupil motivation and satisfaction [51].

### Perceived knowledge gain and opportunities for further learning

Previous research indicates that riding schools are rooted in their traditions [29] and that riding teachers tend to focus on riding technique to the detriment of theory and reflection [31,52]. While the present study does not reveal whether this remains the case, the results suggest a strong desire among teachers to enhance their own subject knowledge and a positive attitude towards basing their teaching on scientific evidence in BW and LC. Hence, most of the teachers wanted to improve their knowledge in BW and LC, and almost all considered it very important to spread evidence-based knowledge in these subjects. This is a very positive finding, as one of the main steps to maintain the equestrian industry’s social licence to operate is through education and promotion of evidence-based practice [53]. Yet, fewer than half of the teachers considered the implementation of evidence-based knowledge in their teaching to be easy, which accentuates the need for the scientific field to translate research into practical terms, communicate its relevance, and support its practical application [54]. Teachers reported that they prefer to gain more knowledge through self-studies such as attendance of clinics, webinars and complementary education. This highlights the potential for enhanced collaboration between researchers, educational institutions, and national equestrian federations.

The results of this study imply that riding schools indeed are significant platforms for knowledge gain. As educational institutions, they are well-positioned to lead the shift toward evidence-based training practices with equine welfare at the core [28]. They can also play a key role in supporting riders in developing their ability to access information via other channels which many riders view as an important, yet often challenging task [55]. Most pupils wanted to improve their own knowledge in BW and LC, mainly by receiving more education at the riding school and gaining increased practical experience. Moreover, pupils most frequently identified their own riding teacher as their main source of knowledge acquisition. Interestingly, both pupils and teachers also commonly specified that common sense or intuition was an important source of learning. Common sense or intuition can be interpreted as tacit knowledge; embodied and implicit knowledge that is challenging to verbally express [56]. Previous research indicates that tacit knowledge is common among equestrians and forms an important part of their expertise [31,57]. However, developing such knowledge takes time and requires time for reflection [31]. Providing a combination of theoretical education, practical experience, and time for reflection is essential in riding school settings. Equally important is that teachers actively reflect on and articulate their own implicit knowledge, so that pupils can more easily learn from them.

### Interest and barriers to teaching and learning

Most pupils were interested in attending separate non-riding lessons in BW and LC, which aligns with previous research. Nyberg et al. [36] investigated Swedish and Finnish riding school pupils’ motivation towards attending non-riding education in BW and LC. Pupils were highly motivated to attend such education (mean score 4.1 on a scale from 1 – strongly disagree to 5 – strongly agree), and they would participate because it is interesting and experienced as important. By contrast, mean scores for unwillingness to participate or amotivation, i.e., the lack of motivation or intention to act, were low (mean score 1.6). Together, these results suggest that the reasons for not attending lessons in BW and LC are not related to a lack of pupils’ motivation or interest, as riding school teachers in the present study seemed to believe. This misalignment in perceptions is further evident in the finding that less than half of the teachers thought that pupils are interested in the subjects, and even fewer believed that pupils want to participate in non-riding lessons. Moreover, the most reported barrier by teachers for providing non-riding lessons in BW and LC was pupils’ lack of interest. Conversely, pupils reported that the biggest obstacle was that such lessons are not provided at all, and a minority cited lack of interest as a barrier. In summary, and contrary to teachers’ beliefs, many pupils expressed a desire for subject education within the school setting, indicating a genuine interest in expanded learning opportunities. Improving the quality of BW and LC teaching may help teachers find greater enjoyment in delivering these lessons and see greater value in the subject, making it easier to meet pupils’ interest and enthusiasm.

Looking beyond interest and motivation, findings from the present study suggest that a lack of time was the second most frequently reported obstacle for subject teaching. This was followed by insufficient educational material and the perception that non-riding lessons are not profitable for the school – factors that teachers consider as significant obstacles to offering such education. Previous research highlights that teachers have several responsibilities at the riding school besides teaching, such as administrative tasks and management of facilities and horses [50]. Allocating time for planning and development of teaching activities may therefore be challenging. Moreover, riding school teachers perceived lack of time during riding lessons tend to result in reduced opportunities for theory and reflection [52]. As both theory and reflection are important for the acquisition of knowledge and skills relating to BW and LC, time constraints should be acknowledged and addressed at riding schools to ensure that pupils have sufficient opportunities to develop a deeper understanding of horses. A better understanding of horse behaviour and welfare is also closely linked to safer human-horse interactions [23], which is one of the focal points of the Swedish Equestrian Federation’s strategy for future riding schools [58].

While lack of availability of educational material was identified by teachers as the third most important barrier for providing non-riding lessons in BW and LC, many of the sources that could potentially help fill this gap were also reported as important sources for knowledge acquisition, i.e., books, equestrian websites and journals, and the Swedish Equestrian Federation. Also, the riding handbooks 1 [59] and 2 [60] were cited as influencing what teachers choose to teach in BW and LC. This raises the question whether there is a shortage of appropriate, evidenced-based and pedagogically designed material that teachers can easily utilise in their teaching of BW and LC. Such educational material could provide teachers with systematic methods and ready-to-use exercises, case studies, and prompts for peer discussion. Such tools can not only support effective knowledge transfer but also help reduce time constraints for teachers. One promising example of a structured framework is the International Society for Equitation Science’s (ISES) 10 Principles of Horse Training [1,27,61], which could serve as a foundation for educational materials that are both evidence-based and practically applicable in riding school settings. The principles are grounded in an acknowledgement of the nature of horses and in an understanding of equine learning theory to promote training methods that are ethical, effective, and supportive of good horse welfare, making them especially well-suited for use in BW and LC education. Examples of research evaluating the practical application and effectiveness of these principles in training and management contexts have already been conducted [27,62,63], including a teaching intervention based on the ISES training principles at two Swedish riding schools [64].

The fourth barrier for providing lessons in BW and LC was that it is perceived as not profitable for the school. Undoubtedly, economics is a concern for any business or organisation. The core activity at riding schools is riding lessons [38,52], and reshaping their structure, for example by integrating more non-riding education, may be perceived as economically unviable or operationally difficult. However, if pupils desire to learn more, which this survey suggests, then providing more than standard riding lessons may offer an economic incentive and a competitive advantage for those riding schools that adopt an alternative structure. In addition to financial concerns, established norms and practices within the riding school culture may also contribute to resistance to change as noted by Lundesjö Kvart [52] and Thorell and Hedenborg [29]. However, economics is not only a limiting factor, it can also serve as a motive for change [50]. Thorell et al. [50] found in their interview study that riding school teachers felt the need to develop their teaching approaches due to economic challenges. In the current context, another key driver for change may be the growing public and industry’s attention to horse welfare and the ongoing discourse around the sport’s social licence to operate [14,53].

Although some pupils experienced no obstacles for attending non-riding lessons, a lack of time still emerged as a common barrier. While this concern was not reported by the majority, it is worth noting that Nyberg et al. [36] similarly found that effort, defined as not having the time and/or energy, was the most common reason for not wanting to participate in non-riding education, despite receiving a modest agreement (mean 2.1 on a 5-point scale). This suggests that time constraints, although not dominant, remain a relevant factor. It may be worth pointing out that the notion of ‘not enough time’ (known as the ‘time paradox’) reflects short-term thinking. In fact, more effective teaching could lead to time savings in the long run. A challenge for the riding school may therefore be to organize the education in ways that allow even time-constrained pupils to participate.

More importantly, however, is perhaps the quality of the education, as only less than half of the pupils were satisfied with the teaching of BW and LC, compared to most of the teachers. Satisfaction of an activity plays an important part in continued participation in leisure [65] and sport activities [66]. For example, experiencing a feeling of purposefulness and euphoria [65] as well as achieving goals [66] have been identified as key factors contributing to satisfaction and re-involvement. Clearly, satisfaction requires involvement in the activity. A noticeable proportion of pupils, compared to relatively few teachers, did not have an opinion on the satisfaction of the teaching. Perhaps these pupils did not feel as being able to judge the quality of the teaching. After all, pupils don’t know what they don’t know [67]. So, even if they want more teaching, they may not know what that could look like. This could also further reinforce the suggestion that many pupils felt that such education is not provided. Thus, some pupils may not be satisfied with the teaching simply because it is not provided.

### Limitations and further research

One important limitation of this study relates to sampling bias, which should be considered when interpreting the results. The target population was riding school teachers and pupils. However, no central registry or complete contact list was available, making it impossible to draw a representative, randomly selected sample. Therefore, data were collected by non-probability sampling [68] and included individuals that had access to, and wanted to take part in the online survey. It is also possible that individuals with a particular interest in the subject were more likely to participate than those with a more neutral attitude. The limited research available, particularly regarding non-riding education and riding school pupils’ experiences further highlights that the results may not be generalizable. Nevertheless, the sample was relatively large and represented different age groups and experience levels of the respondents, as well as locations of riding schools. The female gender was overrepresented, which is in line with gender distribution within the equestrian sport in Sweden [69].

The second potential limitation relates to social desirability bias. It is generally acknowledged that respondents conform to norms and may want to appear ‘good’ and acceptable, i.e., well-informed, active, and responsible [70]. For example, respondents may be more likely to report that they value evidence-based knowledge when participating in research, as that attitude would be socially acceptable in the context. Moreover, respondents may be inclined to report a desire to take part in something that is believed to be preferable [70]. Therefore, intended participation in education does not necessarily correlate with actual participation.

The third potential limitation is inconsistency in respondents’ interpretation of the constructs ‘horse behaviour and welfare’ and ‘horse learning and human-horse communication’. It is expected that respondents understand these constructs differently depending on, e.g., experience level and age. In the present study, we aimed to define these constructs in a broad manner, so that the respondents would be more likely to adopt an inclusive interpretation.

Considering the limitations of this study, future research could address the same research topic with different data collection and sampling methods to build upon the current findings. By utilising probability sampling, representativeness and generalizability could be met to a greater extent. Further, interviews and field observations could add to these results by giving more in-depth information and objective insights. Specifically, an investigation of what is taught and learned regarding BW and LC during different teaching occasions would be of value, as this was one of the topics where teachers and pupils had very different views. While non-riding education in BW and LC is suggested as important, future intervention and longitudinal studies are needed to explore how this education can be effectively structured to support attendance and achievement of meaningful learning outcomes.

## Conclusions

Riding schools play a key role in equestrian education and teachers are well-placed to support the shift toward evidence-based horse training and management. This study highlights the need for improved teaching in the subjects of behaviour and welfare, and learning and communication, particularly through non-riding lessons and clearer alignment between teaching and learning outcomes. Such improvements can help better engage pupils, meet their expectations, and promote the co-creation of meaningful learning experiences. While teachers value evidence-based knowledge, challenges such as perceived time constraints, limited resources, and economic pressures may hinder implementation. Addressing these barriers through improved support, provision of pedagogical materials, and teacher training could strengthen both horse welfare and rider safety and may enhance human-horse interactions overall.
